# A SYSTEMS APPROACH TO PRACTICE TRANSFORMATION FOR BRAIN HEALTH INTEGRATION INTO COMMUNITY HEALTH CENTER SERVICES

**DOI:** 10.1093/geroni/igad104.3155

**Published:** 2023-12-21

**Authors:** Cassie Lindholm, Cheryl Modica

**Affiliations:** National Association of Community Health Centers, Bethesda, Maryland, United States; National Association of Community Health Centers, Bethesda, Maryland, United States

## Abstract

Community Health Centers are America’s largest and most successful primary care system, providing access to quality healthcare services to more than 30 million Americans through more than 14,000 service delivery locations regardless of patients’ ability to pay. The National Association of Community Health Centers (NACHC) provides the training and education that health centers need to advance practice transformation and improve care delivery models to succeed in value-based care and meet the needs of patient populations. Through the Value Transformation Framework (VTF), NACHC provides an approach to support health center systems change, organizes and distills evidence-based interventions, provides knowledge, tools and resources, and links performance to the Quintuple Aim – improved health outcomes, patient and staff experience, reduced costs, and equity. NACHC guides health centers in the application of the VTF through a nation-wide learning community, Elevate. Health center participation in Elevate is linked to improved performance on clinical quality measures, improved patient and staff experiences, and reduced costs. Within the Elevate, NACHC successfully implemented a learning track focused on brain health, offering health center-specific guidance on a clinical introduction to Dementia early detection/risk reduction; incorporating Dementia early detection/risk reduction into care management workflows and leveraging reimbursement opportunities; and strengthening community linkages and partnerships to support Dementia early detection/risk reduction. Evaluation data from this learning track indicates health center interest in continuing education related to brain health integration into health center services. This topic is especially relevant considering the increasing population segment of older adult patients that health centers serve.

